# A case of prenatal diagnosis of meckel-gruber syndrome in one of the dizygotic twin of a naturally conceived pregnancy

**DOI:** 10.1259/bjrcr.20210097

**Published:** 2021-11-03

**Authors:** Akshay Rohatgi, Anupama Tandon

**Affiliations:** 1 Department of Radiology, University College of Medical Sciences and Guru Teg Bahadur Hospital, Delhi, India

## Abstract

Meckel-Gruber syndrome in one twin of a naturally conceived dizygotic twin pregnancy is largely unknown and has not been reported till date. This report illustrates the sonographic features in a case of 20-week twin pregnancy where one twin had an occipital encephalocele, bilateral enlarged and cystic kidneys, hepatic cyst and oligohydramnios but the other twin was normal. The affected twin succumbed within few days after normal vaginal delivery while the normal twin survived and is healthy.

## Background

A 23-year-old female primigravida with twin pregnancy was referred by a gynecologist to our department for anomaly scan (level 2) at around 20 weeks of gestation, which indeed was the first ultrasound scan of her pregnancy. Obstetric history hinted towards discrepancy in the growth parameters of the twins. There was no prior history of miscarriage or consanguinity or any significant family history. Also there were no records of any prior ultrasound scan done. Available investigations included TORCH panel, which was negative.

**Figure 1.**
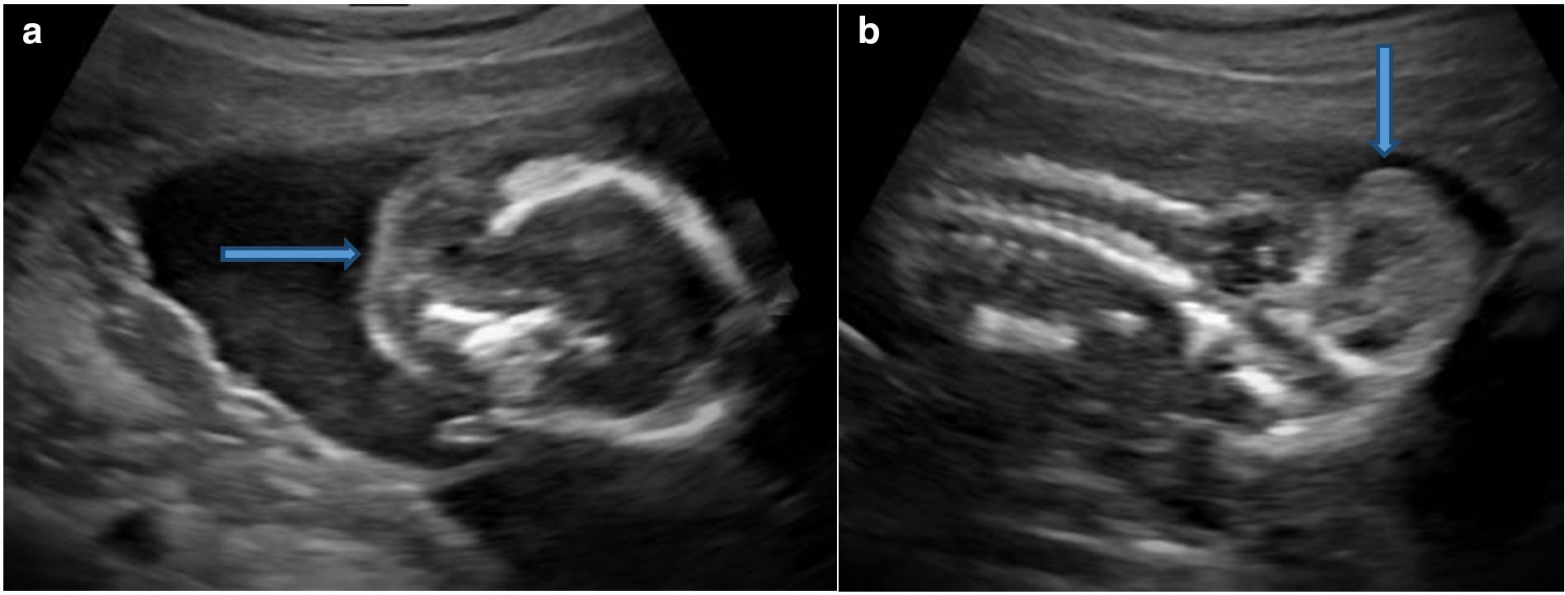
Ultrasound. Axial section (A) and Oblique coronal section (B) of fetal brain showing occipital encephalocele (block arrow).

**Figure 2.**
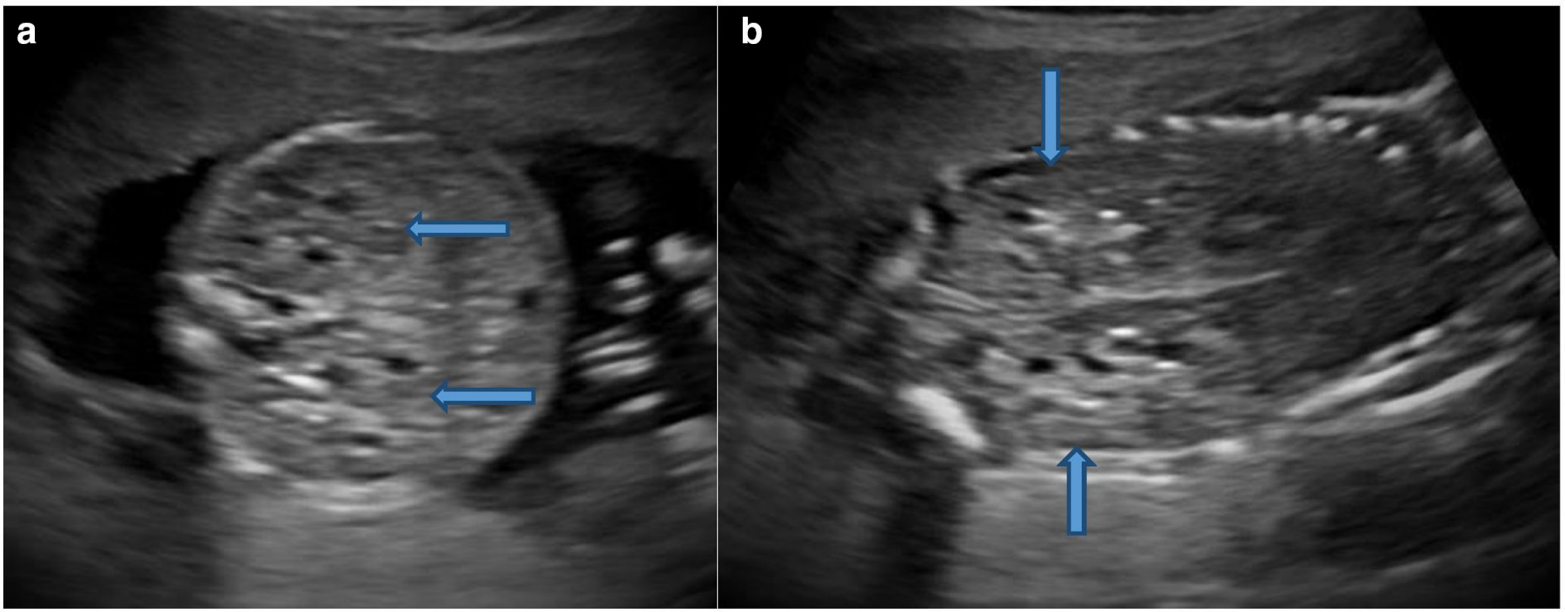
Ultrasound. Axial section (A) and Coronal section (B) of the abdomen showing bilateral enlarged and echogenic kidneys with few small cysts (block arrows).

**Figure 3.**
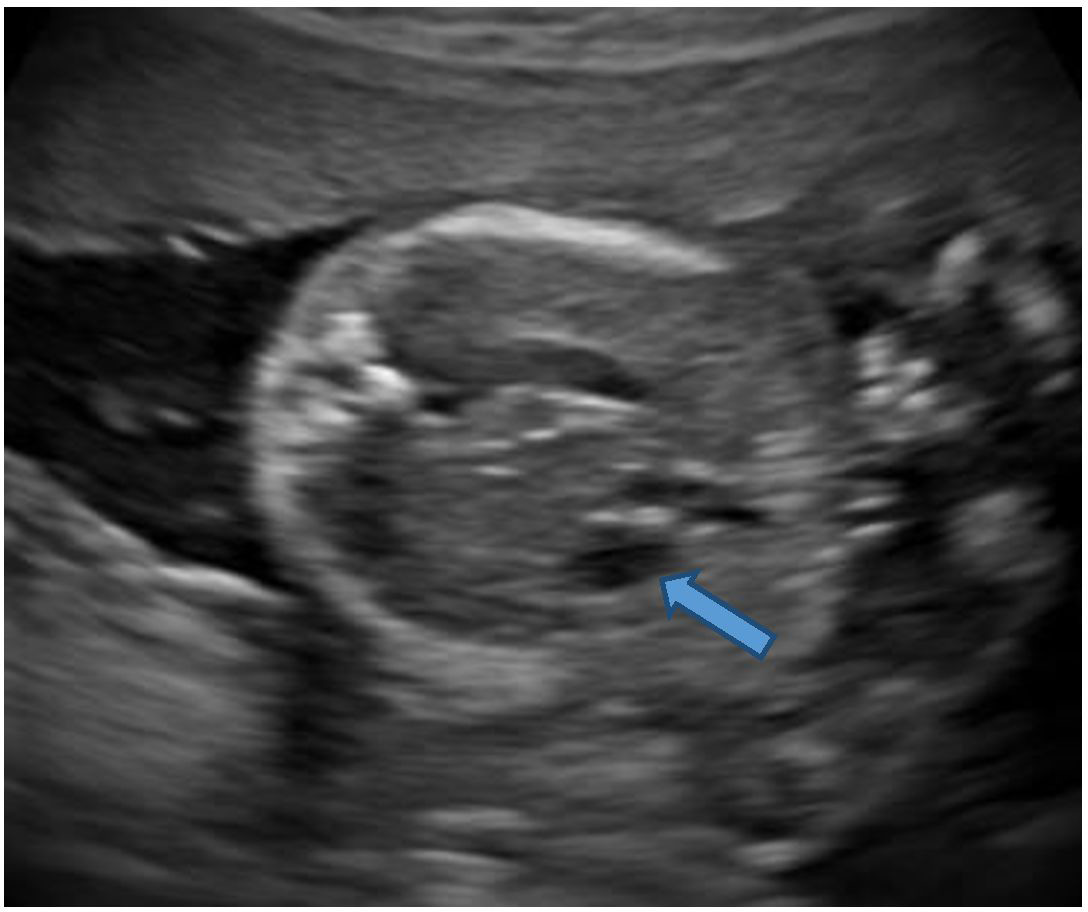
Ultrasound. Axial section of the upper abdomen shows a small hepatic cyst (block arrow).

**Figure 4.**
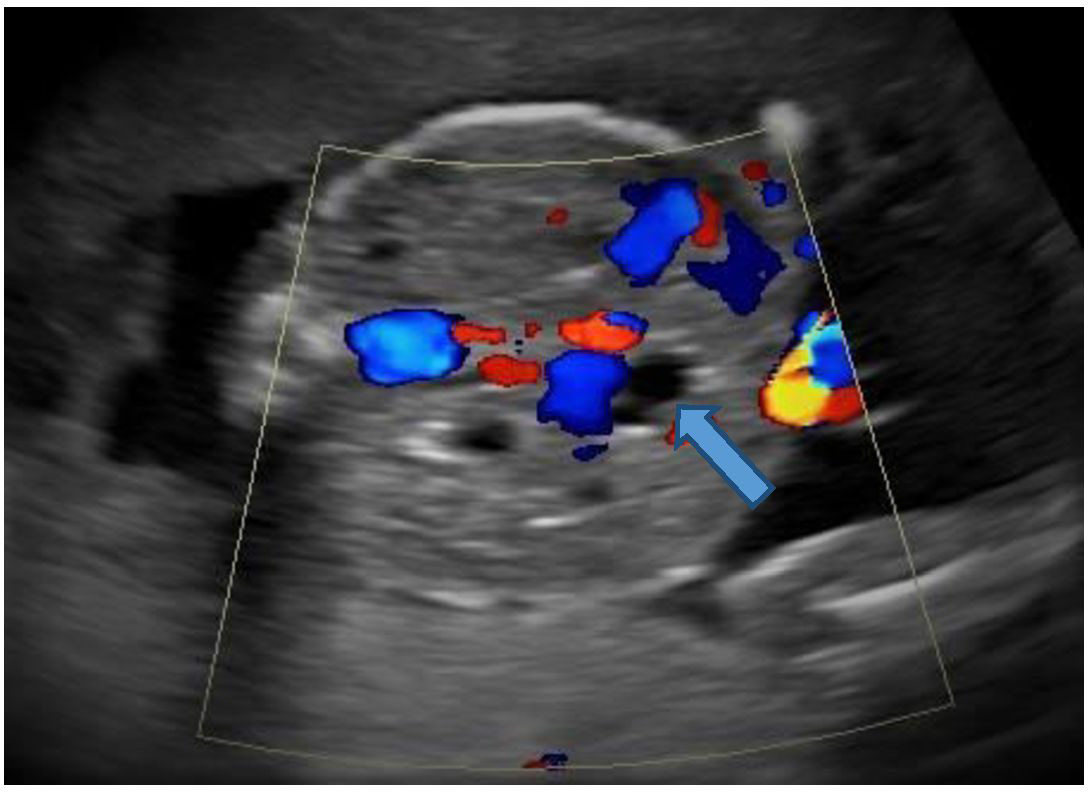
Color Doppler shows no color within the cystic structure (block arrow) in the liver suggesting a hepatic cyst.

The sonography showed twin gestation which was dichorionic and diamniotic in nature. The affected twin had occipital encephalocele with dysmorphic head (Figure 1), reduced biparietal diameter (30 mm) and head circumference (121 mm), bilateral enlarged and cystic kidneys (Figure 2), solitary hepatic cyst (Figures 3 and 4) and decreased femur length (27 mm) for gestation along with oligohydramnios. Whereas the unaffected twin showed no gross congenital anomaly and all the parameters were between 20 and 21 weeks of gestation with adequate liquor for gestation. However, no gross difference was noted in the effective fetal weight on subsequent scans. The patient delivered the twins at around 36 weeks of gestation per vaginally. The affected baby with a birth weight of around 2.3 kg showed signs of failure to thrive and later succumbed 2 days post-delivery, whereas the unaffected baby with a birth weight of around 2.7 kg was normal.

## Discussion

Meckel-Gruber syndrome is a lethal congenital condition having an autosomal recessive pattern of inheritance. It was first described by Johann Friedrich Meckel in 1822 and later by GB Gruber in 1934 who coined the term *‘dysencephalia splanchno cystica’* for this syndrome. This condition is classified as a ‘ciliopathy’ which is characterised by mutations in the genes encoding for the structural proteins of cilia and is considered to be the most severe ciliopathy.

Overall the worldwide incidence varies between 1 in 13,250 and 1 in 140,000 live births, with the highest incidence reported in Gujarati Indians of 1 per 1,300 live births (carrier rate of 1 in 18) by *Young* et al. High incidence was also noted among Belgians and Bedouins in Kuwait (1 in 3,500 with a carrier rate of 1 in 30), Finnish population (1 in 9000 with a carrier rate of 1 in 50). Almost equal incidence was noted in males and females. A high incidence was seen in consanguineous marriages and a recurrence rate of around 25% was noted in each pregnancy. Mortality was nearly 100%.^1^

Over the years, various diagnostic criteria have been defined and refined accordingly. The most consistent finding is bilateral cystic renal dysplasia. Other additional features include posterior fossa abnormalities (like occipital encephalocele, Dandy–Walker malformation), hepatic developmental anomalies (like hepatic cysts, periportal fibrosis) and post-axial polydactyly. Occasional findings include bowing and shortening of the long bones, abnormalities of the male genitalia, microcephaly or anencephaly, cleft lip/palate, congenital heart defects and pulmonary hypoplasia. Uncommon findings include cystic dysplasia of the lungs or thyroid, retinal coloboma and situs defects.^2^

The major criteria comprised of at least 2 out of 3 classic manifestations which included cystic renal dysplasia, occipital encephalocele or any other CNS anomaly and polydactyly which according to *Parelkar et al* had an incidence of around 100%, 90% and 83.3%, respectively.^1^

Over the years with evolution of ultrasound machines, growing knowledge and radiological finesse due importance have been given to the first trimester diagnosis of Meckel-Gruber syndrome, which can be made as early as 12–14 weeks with detection of the aforementioned classic manifestations. This can be complemented by detecting elevated levels of serum alphafetoprotein in maternal blood.

Currently, no recommendations are made illustrating the role of selective termination (intrauterine fetal reduction) via intracardiac injection of potassium chloride (KCl) of the abnormal fetus in Meckel Gruber syndrome. In general, multifetal pregnancy carries higher risk of preterm delivery resulting in a premature neonate who may present with gamut of complications and ensuing postnatal disability. The purpose of selective termination is to reduce the above risk and thereby preventing social, psychological, economic repercussions ensuing later in raising a handicapped child by the parents. However, the decision opting for selective termination would also be influenced by the nature of disease and resulting fetal mortality, risks of procedure etc.^3^ So the need for selective termination may seem unnecessary in diseases carrying very high mortality of the affected fetus. Nevertheless proper patient counselling about the aforementioned should prevail in order to help the patient to arrive at a decision.

After extensive literature search, we came across only one case report by Shozu et al on antenatal detection of Meckel-Gruber syndrome in one of the dizygotic twin but that was following *in vitro* ferilisation and embryo transfer.^4^ Also there were very few case reports like by Juabeh et al^5^ and Shetty et al,^6^ which detected this condition in one of the twins during the postnatal period. To our knowledge, this is the first case report on prenatal diagnosis of Meckel-Gruber syndrome in one of the dizygotic twin following natural conception.

## Learning point

Meckel-Gruber syndrome is a lethal congenital anomaly associated with various anomalies. It is imperant that the radiologist should be able to detect this condition on sonography in the antenatal period paving way for pregnancy termination in case of singleton pregnancy, whereas in the scenario of multiple pregnancies, the pregnancy can be kept under antepartum surveillance as the unaffected fetus is usually unharmed.
